# Mosquito vector competence for Japanese encephalitis virus: a systematic review and meta-analysis update

**DOI:** 10.1186/s13071-025-06843-7

**Published:** 2025-05-26

**Authors:** Stephen Edache, Andrea L. Dixon, Ana R. S. Oliveira, Lee W. Cohnstaedt, Dana Mitzel, Chad E. Mire, Natalia Cernicchiaro

**Affiliations:** 1 Department of Diagnostic Medicine and Pathobiology, Center for Outcomes Research and Epidemiology, College of Veterinary Medicine, Kansas State University, Manhattan, KS USA; 2 Department of Population Health and Reproduction, School of Veterinary Medicine, University of California, Davis, CA USA; 3 Agricultural Research Service, United States Department of Agriculture, National Bio- and Agro-Defense Facility, Manhattan, KS USA

**Keywords:** Japanese encephalitis virus, Flavivirus, Knowledge synthesis, Meta-analysis, Meta-regression, Vector competence

## Abstract

**Background:**

Japanese encephalitis is an emerging zoonotic disease caused by the Japanese encephalitis virus (JEV), transmitted primarily by mosquitoes of the *Culex* species. Amid the recent geographical expansion of JEV into Mainland Australia and the dramatic increase in research output, here we provide an update to our 2018 systematic review and meta-analysis, by appraising the scientific literature published from 2016 through 2023 and quantitatively summarizing the data from this update and the 2018 systematic review meta-analysis on vector competence for JEV.

**Methods:**

A systematic review of the literature on JEV vector and host competence, published from 2016 through 2023, was performed. Bibliographic databases, PubMed, Scopus, Web of Science, and the Armed Forces Pest Management Board website were searched for relevant literature. Records were screened for relevance for vector competence, specifically: infection rate, dissemination rate, and transmission rate. To estimate the overall and subgroup effect sizes for each mosquito species, random-effects meta-analysis models were utilized. Meta-regression models were fit to evaluate the association between a priori variables—such as mosquito subfamily/tribe, routes of JEV administration for mosquito infection, incubation length, incubation temperatures, and diagnostic methods for JEV detection—and the outcomes of interest.

**Results:**

This study update includes 74 new reports, identifying 9–12 additional mosquito species as competent for JEV, depending on the specific outcome assessed. The overall JEV infection, dissemination, and transmission rates across all species and studies were 45.4% (95% confidence interval (CI) 35.9–55.2%), 41.2% (95% CI 29.7–53.7%), and 22.7% (95% CI 14.6–33.4%), respectively. Among the subfamilies/tribes, Culicini had the highest infection (51.9%; 95% CI 39.2–64.4%) and transmission (27.8%; 95% CI 16.5–43.1%) rates. Meta-regressions showed mosquito subfamily/tribe was consistently associated with all the outcomes of interest, although the heterogeneity (*I*^2^) between studies remained consistently high (*I*^2^ > 83.47).

**Conclusions:**

The information presented in this study provides a quantitative summary update on vector competence for JEV. Vector competence data are necessary for risk assessment models, the development of mosquito and virus surveillance programs, and effective prevention and control strategies in regions currently affected by JEV and those at risk of incursion.

**Graphical abstract:**

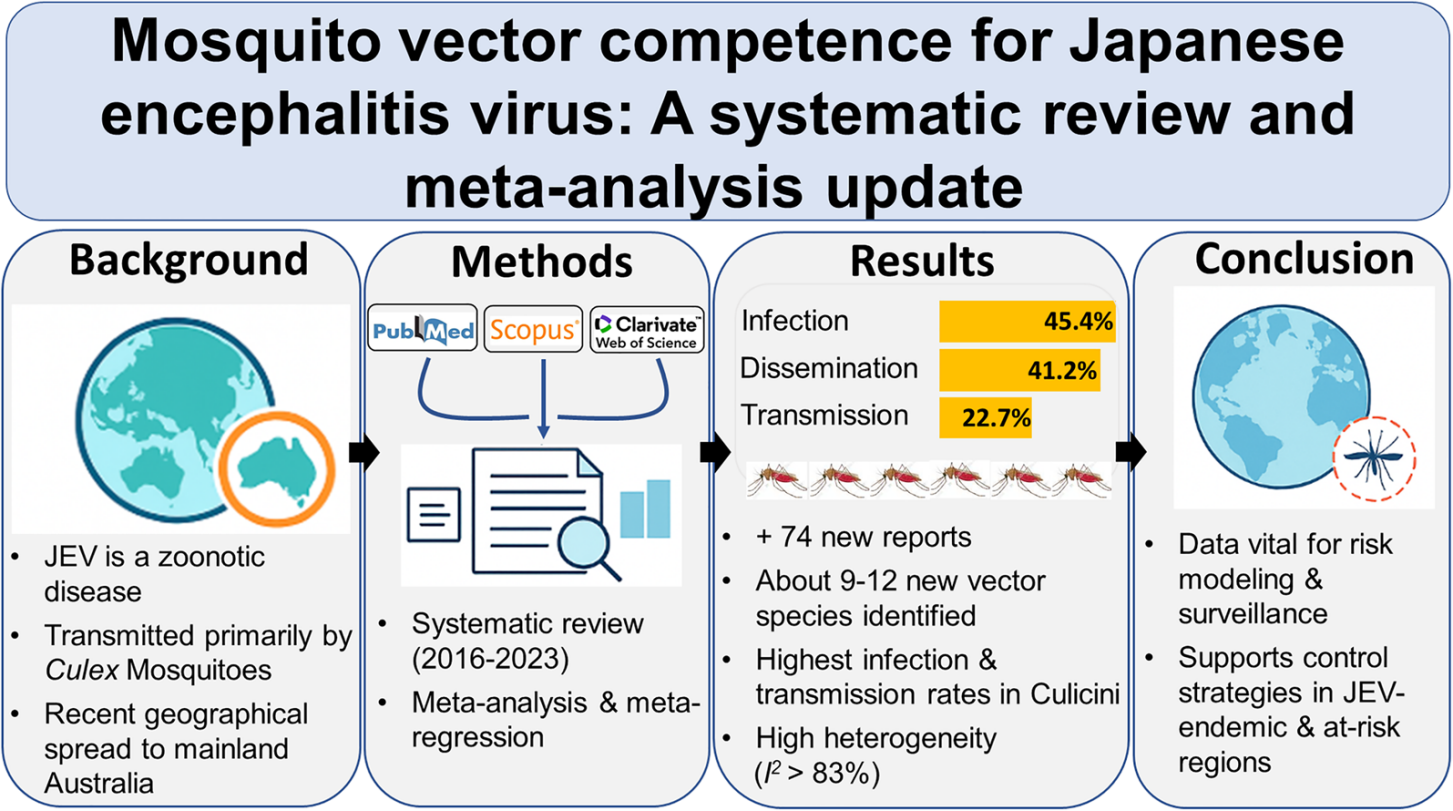

**Supplementary Information:**

The online version contains supplementary material available at 10.1186/s13071-025-06843-7.

## Background

Japanese encephalitis (JE) is an emerging zoonotic disease caused by the Japanese encephalitis virus (JEV), a flavivirus primarily transmitted by mosquitoes of the *Culex* genus. JE is a significant public health concern, causing around 100,000 cases and 25,000 deaths each year globally [1, 2]. Children aged 0–14 years are the most affected, with an incidence rate of 5.4 cases per 100,000 person-years [2]. The disease in humans presents as fever, headache, and respiratory and gastrointestinal symptoms, potentially leading to encephalitis, seizures, coma, and death [3]. Similar clinical signs in combination with reproductive system abnormalities occur in animals, primarily domestic and feral swine, the main amplifying hosts of the virus. The JEV maintains its life cycle between mosquitoes and vertebrate hosts, with swine and *Ardeidae* birds acting as amplifying and reservoir hosts, respectively [4].

JEV is mainly found in 25 countries across eastern and southeastern Asia, including Japan, Thailand, the Philippines, and Indonesia, where infection rates have been consistently maintained in both humans and animals [5]. In contrast, recent data from China show a decline in human infection rates, largely owing to vaccination campaigns and agricultural improvements [6]. As of February 2023, JEV has been isolated from several animal cases, mostly piglets at piggeries and sentinel chickens, and over 40 human cases in Australia, resulting in seven human deaths [6]. Since December 2024, six human infections and one death have been notified, in addition to JEV being detected in mosquitoes and animals in New South Wales, Queensland, Victoria, and Western Australia [7]. While the exact mode of introduction to Australia remains unclear, it is believed to involve several factors, including the presence and persistence of infected mosquito species [6]. Other studies further highlight the importance of mosquito species diversity in sustaining JEV transmission within these regions, with the identification of new species recently shown to be competent for JEV [8]. Understanding vector competence, specifically the rates of infection, dissemination, and transmission of JEV, is crucial to anticipating the spread of the virus and mitigating its impact. As environmental changes, agricultural practices, and vector distributions evolve, updated information is necessary to inform public health strategies and prevent continuous large-scale outbreaks.

While experimental research trials are crucial to demonstrating vector competence of JEV transmission, knowledge synthesis tools, such as systematic reviews and meta-analyses, are essential to inform the agencies and groups responsible for preventing and controlling the introduction of foreign pathogens and responding promptly to potential outbreaks. In 2018, our research team conducted a systematic review and meta-analysis (covering literature from 1950 to 2016) to assess and quantitatively summarize data on vector competence necessary for the maintenance and transmission of JEV [9, 10]. While subsequent reviews [8, 11, 12] have expanded the knowledge on JEV vector infection and competence since our first review, this update of our previous systematic review includes an additional two years of research (2022–2023) beyond the most recent review [8] and is unique in its meta-analyses of the specific JEV vector competence outcomes of infection, dissemination, and transmission rates by species. The objective of this study is to update the 2018 systematic review and meta-analysis [9, 10] by systematically reviewing the literature on vector and host competence for JEV from 2016 through 2023 and summarizing all available data (from both the 2018 review and the current study) on vector competence for JEV worldwide using descriptive tables and meta-analysis and meta-regression models.

## Methods

Although not registered, a protocol developed a priori and all subsequent amendments are published on K-REX [13, 14]. The reporting of this review and meta-analysis follows the Preferred Reporting Items for Systematic reviews and Meta-Analysis (PRISMA) guidelines [15].

### Systematic review of the literature

The overall research question for this systematic review update was: What information is available on vector and host infection and competence for JEV transmission from peer-reviewed literature published from 2016 through 2023? This question informed the search, eligibility criteria, relevance screening, and data extraction steps, but the outcomes included in this meta-analysis and meta-regression pertain only to vector competence for JEV transmission, specifically infection rate, dissemination rate, and transmission rate.

#### Eligibility criteria

We utilized the same inclusion and exclusion criteria as reported in table 1 of the 2018 systematic review [9], with the following minor adjustments: all reports must have been published between 2016 and 2023, be peer-reviewed, and be written in English; the study population had to be mosquito vectors or nonhuman vertebrate hosts of JEV; the outcomes of interest were transmission efficiency, infectiousness, susceptibility to infection, length of incubation, and duration of viremia; and the study types eligible were challenge trials, field studies, observational studies, and experimental studies.

**Table 1:** The databases searched, interfaces used to access the databases, search terms, and the number of references retrieved from the searches performed on 10 January 2024

Database	Interface	Search string^a^	Number of references
Web of Science Core Collection; KCI-Korean Journal Database; MEDLINE; SciELo Citation Index	Web of Science	(TS = ((Japanese encephalitis OR viral encephalitis OR JE OR JEV))) AND TS = (vector competence AND mosquito AND host competence) and English (Languages)^b^	148
PubMed	National Library of Medicine	(Japanese AND encephalitis) AND ((vector OR host) competence) Filters: English, from 2016 to 2023^c^	220
Scopus	Scopus, Elsevier	(TITLE-ABS-KEY(((*vector* AND *competence*) OR (*mosquito* AND *vector* AND *competence*) OR (*host* AND *competence*))) AND PUBYEAR > *2015*) AND (TITLE-ABS-KEY((*"japanese encephalitis"* OR (*viral* AND *encephalitis*) OR *"je"* OR *"jev"*)) AND PUBYEAR > *2015*) AND (LIMIT-TO (LANGUAGE, *"English"*))	50
The Armed Forces Pest Management Board	The Armed Forces Pest Management Board	Find results in AFPMB website (http://www.afpmb.org/content/search-afpmborg) with all the words: Japanese Encephalitis	0

^a^TS = Search for topic terms in the following fields within a record. Search in title, abstract, author keywords, and keywords Plus^®^. TITLE-ABS-KEY = Search for topic terms in the title, abstract, and keywords

^b^This search was manually filtered to only include records from 2016 to 2023

^c^This search string differs from the original—“mosquitoes” was replaced by “(vector OR host) AND competence)”

#### Information sources and search strategy

Table 1 describes the database searches and search strategy used. Three electronic databases, viz. Web of Science, PubMed, and Scopus, were searched on 10 January 2024. Scopus was included in this update as it indexed journals that were previously searched by hand: *The American Journal of Tropical Medicine and Hygiene*, *Journal of the American Mosquito Control Association*, *Journal of Medical Entomology*, and *Vector-Borne and Zoonotic Diseases*. We utilized the same search strategy as the 2018 systematic review [9], except the PubMed search string was changed to be more specific: “mosquitoes” was replaced by “(vector OR host) AND competence)”. The Armed Forces Pest Management Board website was searched by hand using the search feature. In addition, the reference lists of all relevant peer-reviewed systematic and scoping reviews identified through the search were searched by hand for missing references. However, data were not extracted from these reviews, and they were not considered further in the review process.

#### Relevance screening

The records identified from the search were exported as Research Information Systems files into the systematic review software, Covidence (Covidence systematic review software, Veritas Health Innovation, Melbourne, Australia), which was used for relevance screening and the risk of bias assessment. Before the relevance screening, duplicate records were removed using the Covidence deduplication tool. The initial relevance screening tool from the 2018 systematic review (fig. 1 in Ref. [9]) was evaluated for clarity and utility. Two reviewers (A.D. and S.E.) assessed the titles and abstracts of 20% of the records, in duplicate, discussing any discrepancies or questions that arose during the screening process. The screening tool was revised, as necessary, for clarity during this process. Using the adapted screening tool (Supplementary Fig. S1), the titles and abstracts of all records were screened for relevance in duplicate (A.D. and S.E.). Reviewers resolved all conflicts by consensus, consulting a third reviewer (N.C.) whenever consensus could not be reached.

#### Risk of bias assessment

The risk of bias (RoB) assessment was performed separately by two reviewers (A.O. or A.D.) using the framework from the 2018 systematic review (see tables 3 and 4 in Ref. [9]). The assessment focused exclusively on collected data, with no evaluation conducted on missing results.

#### Data collection and data items

A data collection sheet, based on Table 2 from the 2018 systematic review [9], was created in Excel^®^ (Microsoft Corp., Redmond WA, 2021). Key data items relevant to vector competence are reported in Table 2. Data collection from relevant reports at the study level was carried out, in duplicate, by two reviewers (A.D. and S.E.). A report refers to a single article or journal publication, whereas a study denotes a specific trial or investigation within a report. As a result, a single report may contain more than one study. Report authors were not contacted to obtain or confirm data. Unclear or missing information was not captured but rather left blank.

**Table 2:** Key data items extracted, including the outcomes of the systematic review of the literature and explanatory variables tested in the meta-regression models

Outcomes	Data item	Explanation
Vector competence		
Outcomes of the systematic review of the literature	Infection rate	The proportion of JEV-positive mosquitoes after experimental exposure to JEV. The number of JEV-positive mosquitoes and the total number of mosquitoes tested were collected. If authors only reported the proportion/percentage infected, reviewers calculated the number infected using the total number of tested mosquitoes. If the total number of mosquitoes tested was not reported, this outcome was not extracted from those reports
	Dissemination rate	The proportion of mosquitoes with JEV on their legs, irrespective of infection status, after experimental exposure to JEV. The number of positive mosquitoes and the total number of mosquitoes tested were collected. If authors only reported the proportion/percentage infected, reviewers calculated the number positive using the total number of tested mosquitoes. If the total number of mosquitoes tested was not reported, this outcome was not extracted from those reports
	Transmission rate	The proportion of mosquitoes orally exposed that transmitted JEV on refeeding or that contained JEV in their saliva/salivary glands. The number of positive mosquitoes and the total number of mosquitoes tested were collected. If authors only reported the proportion/percentage infected, reviewers calculated the number positive using the total number of tested mosquitoes. If the total number of mosquitoes tested was not reported, this outcome was not extracted from those reports
	Mosquito species	The mosquito species as reported by the author
	Mosquito subfamily/tribe	From the reported species name, reviewers grouped individual species into the subfamily Anophelinae or different tribes within the subfamily Culicinae based on Ref. [18]
Explanatory variables tested in meta-regression models	Administration route	The specific administration route used to experimentally infect mosquitoes with JEV; it comprises intrathoracic inoculation, oral feeding, or vertical transmission
	Length of incubation	The number of days between experimental infection and testing. The outcomes were collected for each reported timepoint. If a range of timepoints were reported, the mean was used. Reviewers categorized the reported period into: ≤ 7 days, 8–14 days, or ≥ 14 days
	Incubation temperature	The temperature of the environment in which mosquito species were kept. The outcomes were collected for each reported temperature. If a range of temperatures was reported, the mean was used. Reviewers categorized the reported temperatures into: ≤ 26 °C, 27–28 °C, or ≥ 28 °C
	Diagnostic method	The diagnostic test used to identify or confirm JEV in vector species: it included polymerase chain reaction (PCR; including real-time reverse transcriptase PCR or reverse transcriptase PCR alone or in combination with antigen capture enzyme assays or virus isolation), virus isolation using cell culture techniques or insect bioassays, or virus isolation using immunofluorescence, hemagglutination inhibition, or neutralization test

### Data analysis

Data synthesis, meta-analyses, and meta-regressions were performed using the R language (version 4.4.1) [16]. Meta-analyses and meta-regressions were carried out using the metafor package (version 4.6-0) [17]. Infection rate, dissemination rate, and transmission rate were synthesized similarly to the 2018 meta-analysis [10], using meta-analysis and meta-regression models with the data from the 2018 systematic review [9] along with the new studies retrieved in this update. Results are presented in alphabetical order based on vector species.

#### Overall and subgroup meta-analysis

For all outcomes, the effect size was calculated as the logit of the proportion of positive mosquitoes/pools to the total number of mosquitoes/pools tested. The value 0.0001 was added to all zero cells. A standard inverse-variance approach random-effects meta-analysis model was fit using the restricted maximum likelihood (REML) method to estimate heterogeneity between studies and the weighted least-squares method to estimate model intercept (i.e., *β*_0_).

As each report could have multiple studies, based on the date/season, location, species, study design, and/or reporting of the populations sampled, we fit a random-effects model with a random slope for study within report, to account for the nonindependence of studies within a single report, and compared it with a random-effects model with a random intercept for study using a likelihood ratio test (LRT). If the LRT was significant (*α* ≤ 0.05), then a random effects model with a random slope for study within report was fit using REML estimation and a *t*-distribution with *k*–*p* degrees of freedom to test individual coefficients and confidence intervals. If the LRT was not significant, a random-effects model with a random intercept for study was fit using REML estimation and the Knapp–Hartung method when multiple studies were available. If only a single study was available, a normal distribution was used to test individual coefficients and confidence intervals. This same method was utilized to determine the best model for the subgroup analyses. Subgroup meta-analyses were performed for each outcome: (1) each mosquito species as reported by the author and (2) by mosquito subfamily/tribe—species were grouped into the subfamily Anophelinae or tribe (e.g., Aedini or Culicini) within the subfamily Culicinae according to Ref. [18], with the exception that all species reported with the genus *Ochlerotatus* were categorized as Aedini.

To quantify the variability among effect sizes that is attributable to heterogeneity, we report the *I*^2^ statistic and utilize the following categorization: 0–40% represents unimportant heterogeneity, 30–60% moderate, 50–90% substantial, and 75–100% considerable heterogeneity [19].

#### Meta-regression

To further investigate the sources of heterogeneity among the studies, a meta-regression model was performed for each outcome, selecting the overall meta-analysis model with the best fit based on the LRT. Included as fixed effects were the predetermined variables of interest (Table 2) as defined in the 2018 meta-analysis [10]. We performed a univariable screen using a cutoff of *P* ≤ 0.10 based on a partial *F*-test. Length of incubation and incubation temperature were originally modeled as continuous variables and tested for linearity against the logit of the outcome; if the linearity assumption was not met, these variables were categorized (Table 2). All significant variables were fit into a multivariable model, if the sample size was large enough; otherwise, bivariable or univariable models were fit, as appropriate, and the effect size and 95% confidence interval are reported.

## Results

### Systematic review of the literature

#### Relevance screening and study characteristics

**Fig. 1:**
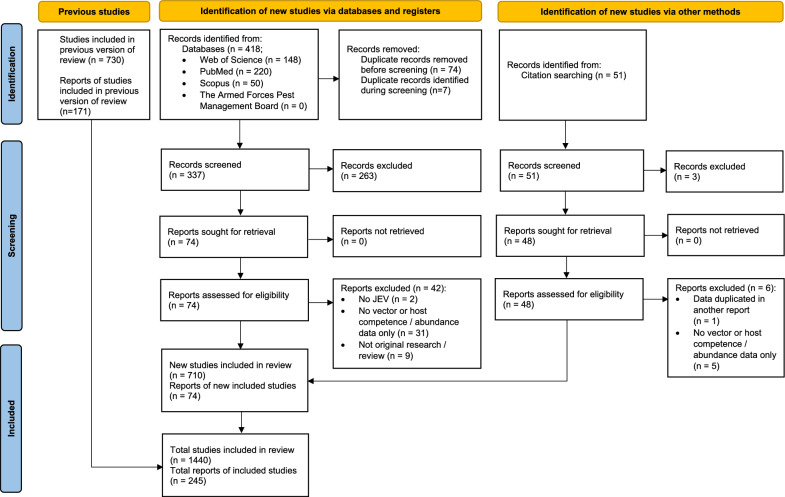
Flowchart of the number of studies and reports identified, screened, and included for data extraction from the 2018 systematic review and current update

A summary of the number of reports and studies at each stage of the systematic review update and the number of included reports and studies from the 2018 review [9] is presented in Fig. 1. Seventy-four new reports were identified. Similar to the 2018 review [9], a majority of reports (66.7%; 26/39) published between 2016 and 2023 were observational, reporting on JEV vector infection, whereas fewer reports (33.3%, 13/39) contained experimental trials focused on both vector infection and competence (Table 3). For completeness and to reflect the full scope of the literature search, similar descriptive data are also reported for host infection and competence in Table 3.

**Table 3:** Source of relevant studies (reports) between 2016 and 2023, by type of outcome, ordered chronologically

Study design	Outcome	Number of studies (reports)	Refs.
Experimental	Vector competence	18 (13)	[21–30, 33–35]
	Host competence	20 (13)	[21, 28, 36–46]
Observational	Vector infection	445 (26)	[12, 47–71]
	Host infection	229 (31)	[40, 47, 59, 60, 67, 68, 72–95]

As this review is focused on vector competence, the subsequent results, including risk of bias assessment and quantitative analyses, are restricted to outcomes pertaining to vector competence. Therefore, 13 reports on vector competence were assessed for risk of bias and included in the meta-analysis and meta-regression, along with 33 additional reports from the 2018 review and meta-analysis [9, 10].

#### Risk of bias (RoB) assessment

The RoB assessment was performed at the report level, and the results are described in Table 4. Overall, all the reports on vector competence (*n* = 13) had a low risk of bias. However, the key domain of randomization—whether it was performed and clearly defined—was not reported in six reports and improperly defined in four reports.

**Table 4:** Risk of bias assessment of all experimental reports (*n* = 13) between 2016 and 2023 that reported a vector competence outcome. Groups are ordered from highest to lowest risk of bias, and references are ordered chronologically within grouping

Number of reports (%)	Bias in key domains				Refs.
	Study population	Study outcomes	Intervention	Randomization	
6 (46.2)	Low risk	Low risk	Low risk	High risk	[21–26]
4 (30.8)	Low risk	Low risk	Low risk	Not defined	[27–30]
2 (15.4)	Low risk	Low risk	Low risk	Low risk	[33, 34]
1 (7.7)	Low risk	Low risk	Low risk	Not applicable	[35]

### Data analysis

#### Infection rate

##### Meta-analysis and subgroup analysis

The JEV infection rate for 47 species was extracted from 377 studies from 29 reports in the 2018 systematic review [9] and 73 studies from 12 reports from this update. The median number of studies per report was 4 (range 1–90). Eleven previously unreported species (*Anopheles plumbeus*, *An. maculipennis freeborni*, *Aedes japonicus japonicus*, *Ae. notoscriptus*, *Ae. varipalpus*, *Ochlerotatus normanensis*, *Oc. purpureus*, *Verrallina carmenti*, *Culiseta annulata*, *Mansonia uniformis*, and *Toxorhynchites splendens*) were included in this meta-analysis. The overall infection rate and infection rates for the subfamily/tribe and individual species are reported in Table 5 and Supplementary Table 1. Table 5 includes newly reported species and those with additional evidence, whereas individual species that did not have any new reports from the 2018 meta-analysis [10] are in Supplementary Table 1.

**Table 5:** Subgroup meta-analysis of studies (*n* = 450) reporting the infection rate of JEV in vectors grouped by either mosquito subfamily/tribe or species

Mosquito subfamily/tribe	Mosquito species	Number of studies (reports)	Infection rate (95% CI)	95% prediction interval	*I*^2^ (%)	ICC (%)
Anophelinae		3 (2)	0.31 (0.20–0.45)	0.19–0.46	2.24	–
	***Anopheles plumbeus***	2 (1)	0.31 (0.04–0.84)	0.03–0.85	4.36	–
	***Anopheles maculipennis freeborni***	1 (1)	0.00 (0.00–1.00)	0.00–1.00	–	–
Culicinae						
Aedini^b^		88 (16)	0.29 (0.18–0.44)	0.02–0.87	85.41	62.73
	*Aedes aegypti*^a^	2 (2)	0.27 (0.20–0.34)	0.20–0.34	0.00	–
	*Aedes albopictus*^a^	35 (8)	0.25 (0.18–0.34)	0.04–0.73	75.34	–
	*Aedes japonicus*^a^	2 (1)	0.90 (0.78–0.96)	0.78–0.96	0.00	–
	***Aedes japonicus japonicus***	8 (2)	0.15 (0.10–0.23)	0.10–0.23	0.00	–
	***Aedes notoscriptus***	1 (1)	0.00 (0.00–1.00)	0.00–1.00	–	–
	***Aedes varipalpus***	1 (1)	0.00 (0.00–1.00)	0.00–1.00	–	–
	*Aedes vexans*^a^	2 (2)	0.04 (0.01–0.15)	0.01–0.15	0.00	–
	*Armigeres subalbatus*^a^	11 (3)	0.37 (0.19–0.58)	0.04–0.89	83.83	–
	*Ochlerotatus detritus*^a^	6 (1)	0.56 (0.34–0.76)	0.12–0.92	58.43	–
	***Ochlerotatus normanensis***	1 (1)	0.00 (0.00–1.00)	0.00–1.00	–	–
	***Ochlerotatus purpureus***	1 (1)	1.00 (0.00–1.00)	0.00–1.00	–	–
	***Verrallina carmenti***	1 (1)	0.00 (0.00–1.00)	0.00–1.00	–	–
Culicini^b^		301 (35)	0.52 (0.39–0.64)	0.04–0.97	92.74	67.94
	*Culex annulirostris*^a^	12 (4)	0.75 (0.54–0.89)	0.19–0.97	53.35	–
	*Culex annulus*^a^	8 (1)	0.42 (0.17–0.73)	0.03–0.95	47.01	–
	*Culex fuscocephala*^a^	4 (1)	0.94 (0.92–0.96)	0.92–0.96	0.00	–
	*Culex gelidus*^a^	5 (3)	0.76 (0.27–0.96)	0.02–1.00	80.47	–
	*Culex pipiens*^a^	31 (6)	0.41 (0.34–0.48)	0.19–0.67	40.22	–
	*Culex pipiens fatigans*^a^	3 (1)	0.16 (0.00–0.91)	0.00–1.00	69.87	–
	*Culex pipiens pallens*^a^	10 (4)	0.15 (0.06–0.35)	0.00–0.86	74.77	–
	*Culex pipiens pipiens*^a^	5 (3)	0.48 (0.12–0.86)	0.01–0.99	89.56	–
	*Culex pseudovishnui*^a^	34 (2)	0.33 (0.25–0.42)	0.06–0.78	85.13	–
	*Culex quinquefasciatus*^a,b^	47 (12)	0.41 (0.17–0.70)	0.01–0.98	96.37	72.39
	*Culex sitiens*^a^	5 (1)	0.87 (0.81–0.92)	0.81–0.92	0.00	-
	*Culex tritaeniorhynchus*^a,b^	104 (15)	0.62 (0.44–0.76)	0.09–0.96	91.15	72.07
	*Culex vishnui*^a^	30 (1)	0.29 (0.22–0.37)	0.08–0.67	78.35	–
Culisetini		8 (2)	0.17 (0.06–0.39)	0.01–0.83	86.20	–
	***Culiseta annulata***	6 (1)	0.28 (0.12–0.52)	0.03–0.83	77.23	–
Mansoniini		4 (1)	0.34 (0.04–0.87)	0.00–1.00	83.68	–
	*Coquillettidia xanthogaster*^a^	2 (1)	0.11 (0.07–0.17)	0.07–0.17	0.00	–
	***Mansonia uniformis***	1 (1)	1.00 (0.00–1.00)	0.00–1.00	–	–
Toxorhynchitini		46 (1)	0.41 (0.37–0.44)	0.37–0.44	0.00	–
	*Toxorhynchites amboinensis*^a^	18 (1)	0.44 (0.39–0.49)	0.39–0.49	0.00	–
	*Toxorhynchites brevipalpis*^a^	10 (1)	0.31 (0.23–0.40)	0.23–0.40	0.00	–
	*Toxorhynchites rutilus*^a^	9 (1)	0.40 (0.35–0.46)	0.35–0.46	0.00	–
	***Toxorhynchites splendens***	5 (1)	1.00 (1.00–1.00)	1.00–1.00	0.00	–
	*Toxorhynchites theobaldi*^a^	4 (1)	0.40 (0.32–0.48)	0.32–0.48	0.00	–
Overall^b^		450 (41)	0.45 (0.36–0.55)	0.04–0.94	90.03	52.62

The number of studies and reports, effect size and 95% confidence interval (CI), 95% prediction interval, heterogeneity (*I*^2^), and intraclass correlation (ICC) for each subgroup model and an overall model are reported. Species not previously included in the 2018 meta-analysis [10] are bolded

^a^Additional studies added since the original meta-analysis [10]

^b^The random effects meta-analysis model, using REML estimation, for the overall estimation and subgroups with an asterisk includes a random slope for study within report; for all other models, a random intercept for study was fit

The overall JEV infection rate across all species and studies was 45.4% (95% CI 35.9–55.2%), with a prediction interval of 4.1–94.2%. The heterogeneity between studies was considerable (*I*^2^ = 90.0%), and about half of the variation was between reports (ICC of 52.6%). The JEV infection rate at the mosquito subfamily/tribe level ranged from 16.8% (Culisetini; 95% CI 6.1–38.5%) to 51.9% (Culicini; 95% CI 39.2–64.4%), and heterogeneity (*I*^2^) ranged from 0% (Toxorhynchitini) to 92.7% (Culicini). Regarding the subgroup analyses at the individual species level, the JEV infection rates for each species ranged from 0% (*Ae. notoscriptus*, *Ae. varipalpus*, *An. maculipennis freeborni*, *Oc. normanensis*, and *Ve. carmenti*; 95% CI 0.0–100.0%) to 100.0% (*Tx. splendens*; 95% CI 100.0–100.0% and *Ma. uniformis* and *Oc. purpureus*; 95% CI 0.0–100.0%). For species with multiple studies (*n* = 32), *I*^2^ ranged from 0% (e.g., *Ae. japonicus japonicus*) to 90.2% (*Cx. pipiens molestus*).

##### Meta-regression

From the univariable meta-regression model screen, mosquito subfamily/tribe (*P* < 0.01) and length of incubation (*P* = 0.07) were significantly associated with JEV infection rates (Supplementary Table 2). From the multivariable meta-regression model, only mosquito subfamily/tribe was significantly associated with the outcome (*P* < 0.01; Table 6). Despite the inclusion of these predictors, the heterogeneity between studies in the multivariable meta-regression was still considerable (*I*^2^ = 90.0%), with just over half of the variation occurring between reports (ICC of 51.2%).

**Table 6:** Multivariable mixed-effect meta-regression model* for the JEV infection rate in vectors (grouped by subfamily) from 114 studies (40 reports). *P*-values, model-adjusted effect sizes, and 95% CI are reported

Variable	Infection rate (95% CI)	*P*-value
Mosquito subfamily/tribe		** < 0.01**
Anophelinae	0.25 (0.03–0.80)^ab^	
Aedini	0.25 (0.15–0.37)^a^	
Culicini	0.53 (0.41–0.64)^b^	
Culisetini	0.36 (0.12–0.71)^ab^	
Mansoniini	0.16 (0.03–0.50)^ab^	
Length of incubation		0.051^#^
≤ 7 days	0.26 (0.14–0.44)	
8–14 days	0.35 (0.20–0.53)	
≥ 14 days	0.28 (0.14–0.47)	

Significant *P*-values (*α* < 0.05) are in bold; Different superscript letters indicate significant pairwise differences at α < 0.05

*I*^2^ = 90.0%; ICC of 51.2%

^#^The *P*-value for the comparison between ≤ 7 days and 8–14 days is 0.07

^*^Standard inverse-variance approach random-effects meta-regression model using the restricted maximum likelihood (REML) method

#### Dissemination rate

##### Meta-analysis and subgroup analysis

Dissemination rate for JEV was reported for 17 mosquito species, from 35 studies from 5 reports from the 2018 systematic review [9] and 51 studies from 10 reports from this update. The median number of studies per report was 4 (range 2–14). Our models included nine species not previously reported in the 2018 meta-analysis [10] (i.e., *An. plumbeus*, *Ae. albopictus*, *Ae. japonicus japonicus*, *Ae. vittatus* (Bigot), *Ar. subalbatus*, *Oc. purpureus*, *Cx. pipiens pipiens,* and *Cx. tritaeniorhynchus*). The overall JEV dissemination rate across all species and studies was 41.2% (95% CI 29.7–53.7%; Table 7) with a 95% prediction interval of 4.5–91.2% and considerable heterogeneity between studies (*I*^2^ = 88.8%).

**Table 7:** Subgroup meta-analysis of studies (*n* = 86) reporting the dissemination rate of JEV in vectors grouped by either mosquito subfamily/tribe or species

Mosquito subfamily/tribe	Mosquito species	Number of studies (reports)	Dissemination rate (95% CI)	95% prediction interval	*I*^2^ (%)	ICC (%)
Anophelinae		2 (1)	0.42 (0.00–1.00)	0.00–1.00	86.48	–
	***Anopheles plumbeus***	2 (1)	0.42 (0.00–1.00)	0.00–1.00	86.48	–
Culicinae						
Aedini*		26 (7)	0.48 (0.22–0.74)	0.04–0.95	84.02	83.98
	***Aedes albopictus***	3 (2)	0.67 (0.61–0.73)	0.61–0.73	0.00	–
	***Aedes japonicus japonicus***	6 (1)	0.15 (0.09–0.25)	0.09–0.25	0.00	–
	***Aedes vittatus*** (Bigot)	4 (1)	0.00 (0.00–0.00)	0.00–0.00	0.00	–
	***Armigeres subalbatus***	1 (1)	0.53 (0.41–0.64)	0.41–0.64	–	–
	***Ochlerotatus purpureus***	1 (1)	0.00 (0.00–1.00)	0.00–1.00	–	–
Culicini*		58 (13)	0.43 (0.27–0.60)	0.04–0.93	90.45	63.00
	*Culex pipiens*^a^	7 (3)	0.31 (0.18–0.48)	0.09–0.66	47.62	–
	***Culex pipiens pipiens***	2 (1)	0.37 (0.00–1.00)	0.00–1.00	94.02	–
	*Culex quinquefasciatus*^a,b^	33 (8)	0.40 (0.23–0.61)	0.05–0.89	88.35	58.82
	***Culex tritaeniorhynchus***	6 (1)	0.92 (0.80–0.97)	0.52–0.99	66.96	–
Overall		86 (15)	0.41 (0.30–0.53)	0.04–0.91	88.77	24.66

The number of studies and reports, effect size and 95% confidence interval (CI), prediction interval, heterogeneity (*I*^2^), and intraclass correlation (ICC) for each subgroup model and an overall model are reported. Species not previously included in the 2018 meta-analysis [10] are bolded

^a^Additional studies added since the 2018 meta-analysis [10]

^b^The random effects meta-analysis model, using REML estimation, for subgroups with an asterisk includes a random slope for study within report; for all other models, a random intercept for study was fit

For the subgroup analyses of the mosquito subfamily/tribes (*n* = 3), the JEV dissemination rates ranged from 42.0% (*Anophelinae*; 95% CI 0–100%) to 47.6% (Aedini; 95% CI 21.5–73.6%) and heterogeneity 84.1% (Aedini) to 90.5% (Culicini) (Table 7). At the individual species level, JEV dissemination rates ranged from 0.0% (*Ae. vittatus*; 95% CI 0.0–0.0%) to 92.2% (*Cx. tritaeniorhynchus*; 95% CI 80.3–97.2%) (Table 7 and Supplementary Table 3). Among species with multiple studies (*n* = 13), heterogeneity ranged from 0.0% (e.g., *Ae. albopictus*) to 94.0% (*Cx. pipiens pipiens*) (Table 7 and Supplementary Table 3).

##### Meta-regression

Only mosquito subfamily/tribe (*P* = 0.09; *I*^2^ = 89.7%) was significantly associated with the JEV dissemination rates in the univariable screen (Table 8), therefore no multivariable models were performed.

**Table 8:** Model-adjusted estimate of the dissemination rate of JEV from univariable meta-regression models^a^

Explanatory variable	Number of studies (reports)	Dissemination rate (95% CI)	*I*^2^ (%)	ICC (%)	*P*-value	Overall *P*-value
Mosquito subfamily/tribe	86 (15)		89.67	41.53		**0.09**
Anophelinae	2 (1)	0.43 (0.06–0.90)			0.94	
Aedini	27 (7)	0.25 (0.12–0.45)			0.03	
Culicini (Reference)	58 (13)	0.46 (0.31–0.62)			–	
Diagnostic method	86 (15)		89.11	28.63		0.72
PCR (Reference)	33 (8)	0.28 (0.07–0.67)			–	
Virus isolation (cell culture techniques or insect bioassays)	41 (6)	0.41 (0.25–0.59)			0.73	
Virus isolation (with immunofluorescence, HAI, or neutralization test)	12 (1)	0.46 (0.26–0.67)			0.54	
Length of incubation	86 (15)		88.77	23.93		0.68
≤ 7 days	30 (9)	0.36 (0.21–0.53)			0.38	
8–14 days (Reference)	47 (15)	0.43 (0.31–0.57)			–	
≥ 14 days	9 (4)	0.39 (0.05–0.89)			0.89	
Incubation temperature	82 (14)		89.11	25.94		0.54
≤ 26 °C	33 (7)	0.41 (0.24–0.59)			0.82	
27–28 °C (Reference)	46 (7)	0.38 (0.23–0.56)			–	
≥ 28 °C	3 (1)	0.66 (0.22–0.93)			0.27	

The explanatory variables and levels, number of studies and reports, measure of heterogeneity, *P*-values, model-adjusted effect sizes, and 95% confidence intervals (CI) are reported. *P*-values of variables that were statistically significant (*α* ≤ 0.10) are in bold

^a^Standard inverse-variance approach random-effects meta-regression model using the restricted maximum likelihood (REML) method

#### Transmission rate

##### Meta-analysis and subgroup analysis

Transmission rates of JEV for 30 species were reported in 89 studies from 15 reports from the 2018 systematic review [9] and 61 studies from 12 reports from this update. The median number of studies per report was 4 (range 1–26). Twelve additional species (*An. plumbeus*, *Ae. japonicus japonicus*, *Ae. vittatus* (Bigot), *Ar. subalbatus*, *Oc. kochi*, *Oc. normanensis*, *Oc. purpureus*, *Opifex fuscus*, *Ve. carmenti*, *Cx. pipiens pipiens*, *Cs. annulata*, and *Ma. uniformis*) that were not previously reported in the 2018 meta-analysis [10] were included in this update. The overall JEV transmission rate across all species and studies was 22.7% (95% CI 14.6–33.4%), with a 95% prediction interval from 1.4% to 85.6% (Table 9). Considerable heterogeneity was observed between studies (*I*^2^ = 84.0%), and less than half of the variation was attributable to differences between reports (ICC of 43.6%).

**Table 9:** Subgroup meta-analysis of studies (*n* = 150) reporting the transmission rate of JEV in vectors grouped by either mosquito subfamily/tribe or species

Mosquito subfamily/tribe	Mosquito species	Number of studies (reports)	Transmission rate (95% CI)	95% prediction interval	*I*^2^ (%)	ICC (%)
Anophelinae		2 (1)	0.03 (0.02–0.07)	0.02–0.07	0.00	-
	***Anopheles plumbeus***	2 (1)	0.03 (0.02–0.07)	0.02–0.07	0.00	-
Culicinae						
Aedini^b^		43 (11)	0.16 (0.07–0.31)	0.02–0.66	59.63	81.13
	*Aedes albopictus*^a^	9 (5)	0.06 (0.02–0.15)	0.00–0.55	46.81	–
	***Aedes japonicus japonicus***	6 (1)	0.12 (0.07–0.20)	0.07–0.20	0.00	–
	***Aedes vittatus*** (Bigot)	7 (1)	0.00 (0.00–0.00)	0.00–0.00	0.00	–
	***Armigeres subalbatus***	1 (1)	0.01 (0.00–0.09)	0.00–0.09	–	–
	***Ochlerotatus kochi***	1 (1)	0.00 (0.00–1.00)	0.00–1.00	–	–
	***Ochlerotatus normanensis***	1 (1)	0.00 (0.00–1.00)	0.00–1.00	–	–
	***Ochlerotatus purpureus***	1 (1)	1.00 (0.00–1.00)	0.00–1.00	–	–
	***Opifex fuscus***	1 (1)	0.00 (0.00–1.00)	0.00–1.00	–	–
	***Verrallina carmenti***	1 (1)	0.00 (0.00–1.00)	0.00–1.00	–	–
Culicini*		95 (22)	0.28 (0.17–0.43)	0.02–0.90	86.39	73.43
	*Culex pipiens*^a^	10 (5)	0.19 (0.08–0.41)	0.01–0.87	76.41	–
	***Culex pipiens pipiens***	4 (2)	0.70 (0.65–0.74)	0.65–0.74	0.00	–
	*Culex quinquefasciatus*^a,b^	27 (7)	0.16 (0.05–0.39)	0.01–0.83	84.94	75.73
	*Culex tritaeniorhynchus*^a,b^	28 (7)	0.42 (0.17–0.72)	0.02–0.96	85.89	71.22
Culisetini		6 (1)	0.22 (0.06–0.54)	0.01–0.91	73.82	–
	***Culiseta annulata***	6 (1)	0.22 (0.06–0.54)	0.01–0.91	73.82	–
Mansoniini		4 (1)	0.25 (0.02–0.82)	0.00–0.99	64.06	–
	***Mansonia uniformis***	1 (1)	1.00 (0.00–1.00)	0.00–1.00	–	–
Overall		150 (27)	0.23 (0.15–0.33)	0.01–0.86	83.98	43.62

The number of studies and reports, effect size and 95% confidence interval (CI), prediction interval, and heterogeneity (*I*^2^) for each subgroup model and an overall model are reported. Species not previously included in the 2018 meta-analysis [10] are bolded

^a^Additional studies added since the 2018 meta-analysis [10]

^b^The random effects meta-analysis model, using REML estimation, for subgroups with an asterisk includes a random slope for study within report; for all other models, a random intercept for study was fit

Results of the subgroup analyses for all subfamily/tribes and species with additional reports and newly reported species are reported in Table 9. Subgroup analyses of species previously reported in the 2018 meta-analysis [10] without additional information are reported in Supplementary Table 4. From the subgroup analyses for mosquito subfamily/tribes (*n* = 5), the JEV transmission rates for these groups ranged from 3.3% (*Anophelinae*; 95% CI 0.2–7.3%) to 27.8% (Culicini; 95% CI 16.5–43.1%). Heterogeneity within mosquito groups ranged from 0% (*Anophelinae*) to 86.4% (Culicini). For the individual species-level subgroup analyses, the JEV transmission rates for each species ranged from 0% (*Op. fuscus*; 95% CI 0–100%) to 99.8% (*Oc. purpureus*; 95% CI 0–100%). For species with multiple studies (*n* = 19), *I*^2^ ranged from 0% (e.g., *Ae. japonicus japonicus*) to 86.0% (*Cx. tritaeniorhynchus*).

##### Meta-regression

From the univariable meta-regression analyses of JEV vector transmission rates, only mosquito subfamily/tribe (*P* < 0.01; *I*^2^ = 83.7%) was significantly associated with the outcome, therefore no multivariable models were performed (Table 10).

**Table 10:** Model-adjusted estimates of the transmission rate of JEV from univariable meta-regression models^a^

Explanatory variables	Number of studies (reports)	Transmission rate (95% CI)	*I*^2^ (%)	ICC (%)	*P*-value	Overall *P*-value
Mosquito subfamily/tribe	150 (27)		83.67	63.60		** < 0.01**
Anophelinae	2 (1)	0.03 (0.00–0.47)			0.13	
Aedini	43 (11)	0.09 (0.04–0.19)			< 0.01	
Culicini (Reference)	95 (22)	0.30 (0.19–0.44)			–	
Culisetini	6 (1)	0.10 (0.01–0.47)			0.20	
Mansoniini	4 (1)	0.18 (0.03–0.57)			0.44	
Administration route	150 (27)		83.71	86.83		0.87
Intrathoracic inoculation (Reference)	3 (1)	0.00 (0.00–1.00)			–	
Oral feeding	147 (27)	0.23 (0.15–0.33)			0.87	
Diagnostic method	147 (26)		84.05	46.82		0.50
PCR (Reference)	37 (10)	0.25 (0.10–0.49)			–	
Virus isolation (cell culture techniques or insect bioassays)	70 (10)	0.17 (0.08–0.33)			0.25	
Virus isolation (with immunofluorescence, HAI, or neutralization test)	40 (6)	0.31 (0.14–0.54)			0.47	
Length of incubation	139 (24)		83.47	39.28		0.87
≤ 7 days	37 (9)	0.20 (0.09–0.37)			0.86	
8–14 days (Reference)	69 (21)	0.19 (0.11–0.30)			–	
≥ 14 days	33 (7)	0.22 (0.10–0.42)			0.61	
Incubation temperature	140 (25)		84.37	43.46		0.62
≤ 26 °C	58 (13)	0.18 (0.09–0.32)			0.51	
27–28 °C (Reference)	68 (10)	0.23 (0.12–0.41)			–	
≥ 28 °C	14 (3)	0.31 (0.10–0.64)			0.65	

The explanatory variables and levels, number of studies and reports, measure of heterogeneity, *P*-values, model-adjusted effect sizes, and 95% confidence intervals (CI) are reported. *P*-values of variables that were statistically significant (*α* ≤ 0.10) are in bold

^a^Standard inverse-variance approach random-effects meta-regression model using the restricted maximum likelihood (REML) method

## Discussion

This systematic review is an update of the 2018 systematic review and meta-analysis of JEV competence of vector species [9, 10]. Similar to the findings in the 2018 systematic review [9], most studies/reports identified in this update that assessed vector infection were observational, whereas only a few, reporting vector competence, were conducted in experimental settings. One advantage of experimental studies lies in their higher internal validity, ensuring the reliability of study outcomes, although observational studies, which are carried out in real-world settings, offer the benefit of higher external validity and greater generalizability of the findings [20]. From the RoB assessment, the experimental reports from this update were generally considered to have a low risk of bias, but 10 of the 13 [21–30] either did not report randomization or improperly defined randomization of the study population (mosquitoes). While this domain is crucial for experimental studies, the nature of the mosquito study population and the design of many entomological experiments often make proper randomization difficult to implement [9].

Moreover, several inherent biases may influence the outcomes of vector competence studies. For instance, many experiments select only mosquitoes that successfully blood-fed, which might inadvertently favor more aggressive individuals and skew results. Laboratory colonies also tend to overestimate vector competence because they often include larger, longer-lived mosquitoes that do not accurately represent natural populations. These laboratory-reared mosquitoes are generally more robust than their wild counterparts, which can lead to inflated estimates. On the other hand, vector competence studies demonstrate a mosquito’s ability to transmit a virus only if it has been exposed to the pathogen. In the wild, exposure depends on the infection status of the host, meaning that even competent mosquitoes may never have the opportunity to transmit if they do not feed on an infected host. While experimental studies ensure exposure by using blood-fed mosquitoes, this does not entirely reflect natural conditions, where both exposure and competence play a role in transmission dynamics.

Despite the research question defined in this study being broad, and data being extracted for several outcomes, here, we report only on the outcomes of infection rate, dissemination rate, and transmission rate of JEV in vector species from experimental studies. These outcomes provide us with relevant information regarding the competence of various mosquito species in JEV transmission. Specifically, dissemination and transmission rates offer more detailed insights into mosquito infectiousness compared with mosquito infection [31]. Notably, some individual mosquito species, particularly species not captured in the 2018 meta-analysis, such as *Oc. purpureus*, *Ma. uniformis*, and *Tx. splendens* (for infection rate only), showed infection and transmission rates reaching up to 100%. However, it is important to interpret these results with caution, as the confidence intervals for these effect sizes range from 0% to 100%, a result of being tested in a single study with low sample sizes. Nonetheless, the quantitative summary provided on these species provides evidence of vector infection beyond the current list of vectors originally known to be JEV competent. With further research studies on these underrepresented species, risk assessment models can be refined to better inform surveillance programs and public health authorities about the increased risk of JEV transmission due to the presence of competent mosquito species.

The overall pooled estimates for JEV infection, dissemination, and transmission rates exhibited substantial to considerable heterogeneity. It is important to recognize that assessing heterogeneity in studies reporting data on mosquito species involves both artifactual and real sources of variability. Artifactual variability arises from differences in study design and other methodological disparities, while real variability, common in entomological studies, stems from the diversity in biological, ecological, and geographical factors, among others, due to the study of multiple and diverse species [19, 20, 32]. Regardless of the source, evaluating and quantifying the causes of heterogeneity enable us to better interpret the pooled mean estimates and their range for these mosquito species [10, 19]. Thus, we evaluated various explanatory variables, including mosquito subfamily/tribe, administration route, diagnostic method, length of incubation, and incubation temperature, for their contributions to the observed heterogeneity in the infection, dissemination, and transmission rates of JEV across mosquito species.

Among these variables, mosquito subfamily/tribe was observed as a significant factor contributing to the heterogeneity across all outcomes of interest in this study. More specifically, the overall infection and transmission rate for Culicini was higher than the overall rates for Aedini. However, these rates were not significantly different from those observed in other subfamilies/tribes such as Anophelinae, Culisetini, and Mansoniini, underscoring the large amount of variability in the estimates and suggesting that these findings should be interpreted with caution. Another predictor variable that contributed to the observed heterogeneity for infection rate is the length of incubation—the period between experimental infection and testing. Although this variable showed marginal significance, it indicated potential variation in JEV infection rates between different incubation lengths. Specifically, in studies where the length of incubation between experimental infection and testing was between 8 and 14 days, mosquito species had higher JEV infection rates compared with those with less than 7 days between infection and testing. Although the length of incubation was not a significant factor for JEV infection rate in the 2018 meta-regression [10], its marginal significance here should not be overlooked, as further research is needed to confirm its association with JEV infection rates in vectors.

Reasons why other explanatory variables, such as administration route, diagnostic method, and incubation temperature, were not associated with the vector competence outcomes could be due to the relative consistency of these variables across reports or the limited number of studies included, which may have prevented the detection of significant statistical associations. Despite accounting for some of the relevant explanatory variables, particularly the different mosquito subfamilies/tribes, substantial heterogeneity in infection, dissemination, and transmission rates persisted across all experimental studies. This suggests that other unmeasured variables could be contributing to this variation. For example, differences in the viral strain (JEV genotype), exposure titers used across studies, frequency of exposure, and environmental factors, such as relative humidity and fluctuating temperatures, could also be major contributors to the observed heterogeneity [4, 11]. While it would be relevant to explore these potential sources of variability, some of these data were infrequently reported across studies, preventing their inclusion in our meta-regression models [16]. Moreover, the exclusion of non-English-language reports represents a limitation that could have restricted access to relevant information on JEV vector competence and infection. This exclusion raises the possibility that critical information was missed, which might have narrowed the confidence intervals of the effect estimates and added additional insights, particularly for underrepresented species. While the precise impact of excluding non-English-language reports on our overall findings remains uncertain, their inclusion could potentially have substantially influenced and enhanced the robustness of results.

Despite these limitations, the data gathered and summarized in this update hold significant value. Specifically, the vector infection rate data can guide surveillance programs to monitor mosquito species, including newly reported ones, with higher JEV infection rates. These mosquito species can act as indicators of JEV circulation and, depending on their contributions to the endemic/epidemic/epizootic JEV cycles, may be confirmed as primary or secondary vectors in JEV transmission. However, in addition to vector infection rate, it is also crucial to have a good understanding of mosquito abundance, host preference, longevity, geographical ranges, and co-occurrences with human settlements or animal reservoirs. Moreover, data on JEV dissemination and transmission rates in vectors are crucial for assessing the risk of JEV introduction and transmission within new geographical areas previously free of the virus. Additionally, these data can assist in understanding the contributions of each competent mosquito species to the overall burden of JEV transmission within specific regions. Thus, identifying JEV-competent mosquito species can help implement targeted mitigation strategies against these species, thereby lowering the number of JEV cases in affected regions and consequently reducing the risk of its spread to susceptible areas.

## Conclusions

While the expansion of JEV into Mainland Australian served as one of the motivating factors for this review update, its primary rationale extends beyond this event. This review provides updated data not only on newly reported species showing competence for JEV but also on the level of competence observed in previously identified species. These findings offer valuable insights that go beyond what individual studies can provide, helping to synthesize the evidence base in a way that is accessible and actionable for policymakers, public health authorities, and disease modelers. Ultimately, our goal is to support evidence-based decision-making with the most current and comprehensive data available.

## Supplementary Information

Supplementary Material 1: Figure S1. Flowchart of relevance screening adapted from the 2018 systematic review [9]. Table S1. Subgroup meta-analysis of studies reporting the pooled estimates of the infection rate of JEV for individual mosquito species from the 2018 meta-analysis [10], with no new studies from this update. The number of studies and reports, measure of effect size and 95% confidence interval (CI), prediction interval, and heterogeneity (*I*^2^) are reported. Table S2. Model-adjusted estimates of the infection rate of JEV from univariable meta-regression models. The predictor and levels, number of studies and reports, measure of heterogeneity, *P*-values, model-adjusted effect sizes, and 95% confidence interval (CI) are reported. *P*-values from explanatory variables that were significant (*α* ≥ 0.1) are in bold. Table S3. Subgroup meta-analysis of studies reporting the pooled estimates of the dissemination rate of JEV for individual mosquito species from the 2018 meta-analysis [10], with no new studies from the updated systematic review. The number of studies and reports, measure of effect size and 95% confidence interval (CI), prediction interval, and heterogeneity (*I*^2^) are reported. Table S4. Subgroup meta-analysis of studies reporting the pooled estimates of the transmission rate of JEV in mosquito species from the 2018 meta-analysis [10], with no new studies from this update. The number of studies and reports, measure of effect size and 95% confidence interval (CI), prediction interval, and heterogeneity (*I*^2^) are reported.

Supplementary Material 2: PRISMA 2020 checklist.

## Data Availability

The data supporting the conclusions of this article are included within the article and its additional files. Raw data are available from the corresponding author upon reasonable request.

## Abbreviations

JE: Japanese encephalitis
JEV: Japanese encephalitis virus
HAI: Hemagglutination inhibition tests
PCR: Polymerase chain reaction
REML: Restricted maximum likelihood
PRISMA: Preferred Reporting Items for Systematic Reviews and Meta-Analysis
