# Supplementary material for: Mosquito vector competence for Japanese encephalitis virus: a systematic review and meta-analysis update
Source: Parasit Vectors. 2025 May 26;18:191. doi: 10.1186/s13071-025-06843-7 (PMC12107883; doi:10.1186/s13071-025-06843-7)
Supplement: Supplementary file 1 — Supplementary Material 1: Figure S1. Flowchart of relevance screening adapted from the 2018 systematic review [9]. Table S1. Subgroup meta-analysis of studies reporting the pooled estimates of the infection rate of JEV for individual mosquito species from the 2018 meta-analysis [10], with no new studies from this update. The number of studies and reports, measure of effect size and 95% confidence interval (CI), prediction interval, and heterogeneity (I2) are reported. Table S2. Model-adjusted estimates of the infection rate of JEV from univariable meta-regression models. The predictor and levels, number of studies and reports, measure of heterogeneity, P-values, model-adjusted effect sizes, and 95% confidence interval (CI) are reported. P-values from explanatory variables that were significant (α ≥ 0.1) are in bold. Table S3. Subgroup meta-analysis of studies reporting the pooled estimates of the dissemination rate of JEV for individual mosquito species from the 2018 meta-analysis [10], with no new studies from the updated systematic review. The number of studies and reports, measure of effect size and 95% confidence interval (CI), prediction interval, and heterogeneity (I2) are reported. Table S4. Subgroup meta-analysis of studies reporting the pooled estimates of the transmission rate of JEV in mosquito species from the 2018 meta-analysis [10], with no new studies from this update. The number of studies and reports, measure of effect size and 95% confidence interval (CI), prediction interval, and heterogeneity (I2) are reported. [file 13071_2025_6843_MOESM1_ESM.docx]

**Supplementary materials for:**

**Mosquito vector competence for Japanese encephalitis virus: A systematic review and meta-analysis update**

Stephen Edache, Andrea L. Dixon, Ana R.S. Oliveira, Lee W. Cohnstaedt, Dana Mitzel, Chad E. Mire, Natalia Cernicchiaro


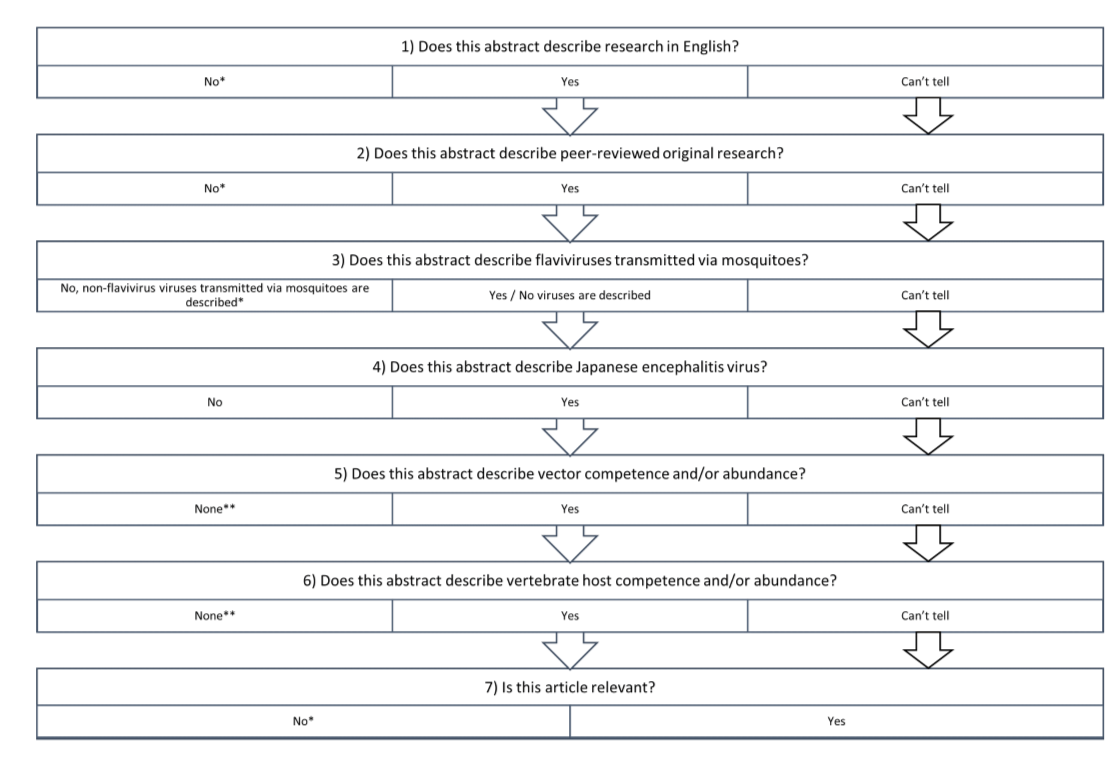


**Suppl. Figure 1**. Flowchart of relevance screening adapted from the 2018 systematic review [1]. (*) If the answer to question 1-4 or 7 was “No”, the record was excluded. (**) If the answers to question 5 and 6 were “None”, the record was excluded.

**Suppl. Table 1.** Sub-group meta-analysis of studies reporting the pooled estimates of the infection rate of JEV for individual mosquito species from the 2018 meta-analysis [2], with no new studies from this update. The number of studies and reports, measure of effect size and 95% confidence interval (CI), prediction interval, and heterogeneity (*I*^2^) are reported.

| Mosquito species | Number of studies (reports) | Infection rate  (95% CI) | Prediction interval | *I*^2^ (%) |
| --- | --- | --- | --- | --- |
| *Aedes alcasidi* | 1 (1) | 0.29 (0.07 – 0.67) | 0.07 – 0.67 | - |
| *Aedes dorsalis* | 1 (1) | 0.03 (0.00 – 0.20) | 0.00 – 0.20 | - |
| *Aedes nigromaculis* | 1 (1) | 0.02 (0.01 – 0.05) | 0.01 – 0.05 | - |
| *Aedes togoi* | 4 (1) | 0.44 (0.21 – 0.70) | 0.21 – 0.70 | 0.00 |
| *Aedes vexans nipponii* | 1 (1) | 0.45 (0.25 – 0.66) | 0.25 – 0.66 | - |
| *Armigeres flavus* | 1 (1) | 0.07 (0.01 – 0.35) | 0.01 – 0.35 | - |
| *Ochlerotatus kochi* | 1 (1) | 0.21 (0.10 – 0.40) | 0.10 – 0.40 | - |
| *Ochlerotatus notoscriptus* | 2 (1) | 0.26 (0.09 – 0.58) | 0.09 – 0.58 | 0.00 |
| *Ochlerotatus vigilax* | 3 (1) | 0.23 (0.06 – 0.59) | 0.03 – 0.73 | 27.91 |
| *Opifex fuscus* | 1 (1) | 0.74 (0.60 – 0.84) | 0.60 – 0.84 | - |
| *Verrallina funereal* | 1 (1) | 0.57 (0.46 – 0.68) | 0.46 – 0.68 | - |
| *Culex pipiens molestus* | 2 (2) | 0.05 (0.00 – 1.00) | 0.00 – 1.00 | 90.17 |
| *Culex tarsalis* | 1 (1) | 0.01 (0.00 – 0.05) | 0.00 – 0.05 | - |
| *Culiseta incidens* | 1 (1) | 0.04 (0.01 – 0.12) | 0.01 – 0.12 | - |
| *Culiseta inornata* | 1 (1) | 0.04 (0.01 – 0.11) | 0.0.1 – 0.11 | - |
| *Mansonia septempunctata* | 1 (1) | 0.67 (0.46 – 0.82) | 0.46 – 0.82 | - |

Random effects sub-group meta-analyses models for the individual species included a random intercept for study and used REML and a Knapp-Hartung estimation procedure. If only one study was available, a Z-test was used instead of the Knapp-Hartung estimation.

**Suppl. Table 2.** Model-adjusted estimates of the infection rate of JEV from univariable meta-regression models. The predictor and levels, number of studies and reports, measure of heterogeneity, *P*-values, model-adjusted effect sizes, and 95% confidence interval (CI) are reported. *P*-values from explanatory variables that were significant (α ≥ 0.1) are in bold.

| Variable | Number of studies (reports) | Infection rate (95% CI) | *I*^2^ (%) | *P*-value | Overall *P*-value |
| --- | --- | --- | --- | --- | --- |
| **Mosquito subfamily/tribe** | 450 (41) |  | 89.43 |  | **< 0.01** |
| *Anophelinae* | 3 (2) | 0.30 (0.03 – 0.84) |  | 0.47 |  |
| Aedini | 88 (16) | 0.30 (0.20 – 0.43) |  | **< 0.01** |  |
| Culicini (Reference) | 301 (35) | 0.52 (0.42 – 0.62) |  | - |  |
| Culisetini | 8 (2) | 0.38 (0.12 – 0.72) |  | 0.44 |  |
| Mansoniini | 4 (1) | 0.18 (0.04 – 0.55) |  | 0.06 |  |
| Toxorhynchitini | 46 (1) | 0.40 (0.07 – 0.86) |  | 0.69 |  |
| **Administration route** | 450 (41) |  | 89.70 |  | 0.21 |
| Intrathoracic inoculation (Reference) | 64 (3) | 0.55 (0.28 – 0.80) |  | - |  |
| Oral feeding | 339 (36) | 0.48 (0.38 – 0.58) |  | 0.61 |  |
| Vertical transmission | 47 (5) | 0.25 (0.10 – 0.51) |  | 0.11 |  |
| **Diagnostic method** | 450 (41) |  | 89.83 |  | 0.21 |
| PCR (Reference) | 74 (14) | 0.46 (0.30 – 0.63) |  | - |  |
| Virus isolation (cell culture techniques or insect bioassays) | 156 (13) | 0.33 (0.19 – 0.51) |  | 0.27 |  |
| Virus isolation (with immunofluorescence, HAI, or neutralization test) | 220 (14) | 0.54 (0.38 – 0.69) |  | 0.53 |  |
| **Length of incubation** | 341 (34) |  | 91.11 |  | **0.07** |
| ≤ 7 days | 123 (17) | 0.41 (0.29 – 0.54) |  | **0.04** |  |
| 8 – 14 days (Reference) | 156 (29) | 0.51 (0.39 – 0.62) |  | **-** |  |
| ≥ 14 days | 62 (12) | 0.41 (0.27 – 0.58) |  | 0.19 |  |
| **Incubation temperature** | 394 (38) |  | 91.52 |  | 0.74 |
| ≤ 26 °C | 133 (19) | 0.48 (0.35 – 0.62) |  | 0.49 |  |
| 27 – 28 °C (Reference) | 222 (16) | 0.42 (0.28 – 0.57) |  | - |  |
| ≥ 28 °C | 39 (6) | 0.51 (0.29 – 0.72) |  | 0.50 |  |

Standard inverse-variance approach random-effects meta-regression model using the restricted maximum likelihood (REML) method.

**Suppl. Table 3.** Sub-group meta-analysis of studies reporting the pooled estimates of the dissemination rate of JEV for individual mosquito species from the 2018 meta-analysis [2], with no new studies from the updated systematic review. The number of studies and reports, measure of effect size and 95% confidence interval (CI), prediction interval, and heterogeneity (*I*^2^) are reported.

| Mosquito species | Number of studies (reports) | Dissemination rate (95% CI) | Prediction interval | I^2^ (%) |
| --- | --- | --- | --- | --- |
| *Ochlerotatus detritus* | 6 (1) | 0.76 (0.54 – 0.90) | 0.20 – 0.98 | 46.40 |
| *Ochlerotatus notoscriptus* | 2 (1) | 0.08 (0.05 – 0.13) | 0.05 – 0.13 | 0.00 |
| *Ochlerotatus vigilax* | 2 (1) | 0.25 (0.00 – 1.00) | 0.00 – 1.00 | 62.14 |
| *Opifex fuscus* | 1 (1) | 0.70 (0.54 – 0.82) | 0.54 – 0.82 | - |
| *Culex annulirostris* | 5 (2) | 0.40 (0.10 – 0.80) | 0.01 – 0.97 | 88.61 |
| *Culex gelidus* | 1 (1) | 0.23 (0.16 – 0.32) | 0.16 – 0.32 | - |
| *Culex sitiens* | 4 (1) | 0.19 (0.05 – 0.49) | 0.02 – 0.71 | 50.73 |

Random effects sub-group meta-analyses models for the individual species included a random intercept for study and used REML and a Knapp-Hartung estimation procedure. If only one study was available a Z-test was used instead of the Knapp-Hartung estimation.

**Suppl. Table 4.** Sub-group meta-analysis of studies reporting the pooled estimates of the transmission rate of JEV in mosquito species from the 2018 meta-analysis [2], with no new studies from this update. The number of studies and reports, measure of effect size and 95% confidence interval (CI), prediction interval, and heterogeneity (*I*^2^) are reported.

| Mosquito species | Number of studies (reports) | Transmission rate (95% CI) | Prediction interval | *I*^2^ (%) |
| --- | --- | --- | --- | --- |
| *Aedes aegypti* | 1 (1) | 0.25 (0.16 – 0.37) | 0.16 – 0.37 | - |
| *Aedes japonicus* | 2 (1) | 0.75 (0.41 – 0.93) | 0.41 – 0.93 | 0.00 |
| *Aedes vexans nipponii* | 1 (1) | 0.33 (0.08 – 0.73) | 0.08 – 0.73 | - |
| *Ochlerotatus detritus* | 6 (1) | 0.33 (0.12 – 0.64) | 0.04 – 0.86 | 60.03 |
| *Ochlerotatus notoscriptus* | 2 (1) | 0.25 (0.03 – 0.75) | 0.03 – 0.75 | 0.00 |
| *Ochlerotatus vigilax* | 2 (1) | 0.12 (0.04 – 0.32) | 0.04 – 0.32 | 0.00 |
| *Verrallina funerea* | 1 (1) | 0.17 (0.05 – 0.41) | 0.05 – 0.41 | - |
| *Culex annulirostris* | 5 (2) | 0.47 (0.17 – 0.79) | 0.03 – 0.96 | 70.36 |
| *Culex fuscocephala* | 4 (1) | 0.16 (0.09 – 0.27) | 0.09 – 0.27 | 0.00 |
| *Culex gelidus* | 10 (4) | 0.33 (0.19 – 0.51) | 0.05 – 0.83 | 82.96 |
| *Culex pipiens molestus* | 3 (1) | 0.80 (0.72 – 0.86) | 0.72 – 0.86 | 0.00 |
| *Culex sitiens* | 4 (1) | 0.24 (0.02 – 0.82) | 0.00 – 0.99 | 75.33 |
| *Coquillettidia xanthogaster* | 2 (1) | 0.07 (0.03 – 0.14) | 0.03 – 0.14 | 0.00 |
| *Mansonia septempunctata* | 1 (1) | 0.54 (0.35 – 0.72) | 0.35 – 0.72 | - |

Random effects sub-group meta-analyses models for the individual species included a random intercept for study and used REML and a Knapp-Hartung estimation procedure. If only one study was available a Z-test was used instead of the Knapp-Hartung estimation.

**References**

1. Oliveira ARS, Strathe E, Etcheverry L, et al. Assessment of data on vector and host competence for Japanese encephalitis virus: A systematic review of the literature. *Prev Vet Med*. 2018;154:71-89. doi:10.1016/j.prevetmed.2018.03.018

2. Oliveira ARS, Cohnstaedt LW, Strathe E, et al. Meta-Analyses of Japanese encephalitis virus infection, dissemination, and transmission rates in vectors. *Am J Trop Med Hyg*. 2018;98(3):883-890. doi:10.4269/ajtmh.17-0622
